# Gene Expression Profiling of Dendritic Cells in Different Physiological Stages under *Cordyceps sinensis* Treatment

**DOI:** 10.1371/journal.pone.0040824

**Published:** 2012-07-19

**Authors:** Chia-Yang Li, Chi-Shiun Chiang, Wei-Chung Cheng, Shu-Chi Wang, Hung-Tsu Cheng, Chaang-Ray Chen, Wun-Yi Shu, Min-Lung Tsai, Ruey-Shyang Hseu, Cheng-Wei Chang, Chao-Ying Huang, Shih-Hua Fang, Ian C. Hsu

**Affiliations:** 1 Department of Biomedical Engineering and Environmental Sciences, National Tsing Hua University, Hsinchu, Taiwan; 2 Department of Urology, University of Texas Southwestern Medical Center, Dallas, Texas, United States of America; 3 Division of Pediatric Neurosurgery, Neurological Institute, Taipei Veterans General Hospital, Taipei, Taiwan; 4 Institute of Nanoengineerin and Microsystem, National Tsing Hua University, Hsinchu, Taiwan; 5 Institute of Statistics, National Tsing Hua University, Hsinchu, Taiwan; 6 Department of Biochemical Science and Technology, National Taiwan University, Taipei, Taiwan; 7 Institute of Athletics, National Taiwan Sport University, Taichung, Taiwan; College of Tropical Agriculture and Human Resources, University of Hawaii, United States of America

## Abstract

*Cordyceps sinensis* (CS) has been commonly used as herbal medicine and a health supplement in China for over two thousand years. Although previous studies have demonstrated that CS has benefits in immunoregulation and anti-inflammation, the precise mechanism by which CS affects immunomodulation is still unclear. In this study, we exploited duplicate sets of loop-design microarray experiments to examine two different batches of CS and analyze the effects of CS on dendritic cells (DCs), in different physiology stages: naïve stage and inflammatory stage. Immature DCs were treated with CS, lipopolysaccharide (LPS), or LPS plus CS (LPS/CS) for two days, and the gene expression profiles were examined using cDNA microarrays. The results of two loop-design microarray experiments showed good intersection rates. The expression level of common genes found in both loop-design microarray experiments was consistent, and the correlation coefficients (Rs), were higher than 0.96. Through intersection analysis of microarray results, we identified 295 intersecting significantly differentially expressed (SDE) genes of the three different treatments (CS, LPS, and LPS/CS), which participated mainly in the adjustment of immune response and the regulation of cell proliferation and death. Genes regulated uniquely by CS treatment were significantly involved in the regulation of focal adhesion pathway, ECM-receptor interaction pathway, and hematopoietic cell lineage pathway. Unique LPS regulated genes were significantly involved in the regulation of Toll-like receptor signaling pathway, systemic lupus erythematosus pathway, and complement and coagulation cascades pathway. Unique LPS/CS regulated genes were significantly involved in the regulation of oxidative phosphorylation pathway. These results could provide useful information in further study of the pharmacological mechanisms of CS. This study also demonstrates that with a rigorous experimental design, the biological effects of a complex compound can be reliably studied by a complex system like cDNA microarray.

## Introduction

*Cordyceps sinensis* (CS) is a species of parasitic fungus on the larvae of the Lepidoptera, and has been commonly used as herbal medicine and a health supplement in China for approximately two thousand years [1]–[3]. Numerous pharmacological effects of CS have been reported such as anti-tumor [4], [5], immunomodulatory [6]–[8], anti-inflammatory [9]–[11], and anti-oxidant properties [12], [13]. Furthermore, CS possesses both suppressive and enhancive properties with regard to human immunity, which could be a reference to the Yin-Yang characteristics of CS described in traditional Chinese medicine [14]. Several reports have evinced this dual modality from the immunological and pharmacological perspective [7], [15]–[17]. In addition, CS contains various bioactive compounds, including cordycepin, adenosine, adenine, guanosine, ergosterol, uridine, uracil, hypoxanthine, mannitol, and polysaccharides [2], [18]. Multiple compound-based drugs may provide important combination therapies that simultaneously influence multiple pharmacological targets and provide clinical efficacy beyond that of single compound-based drugs [19].

Microarray technology is being applied widely to address increasingly complex scientific questions [20]. Microarray experiments yield lists of tens or hundreds of differentially regulated genes in sets of experiments. However, the presence of dissimilar regulatory patterns among functionally related genes makes it difficult for the biological interpretation of microarray data [21]. This is not surprising, because systematic biases and random variations are inherent in microarray data [20]. A careful experimental design and rigorous statistical analysis can increase the precision of microarray measurements [22], [23]. Moreover, statistical assessment is not only important in data analysis, but also plays a critical role in every stage of the microarray investigative process, including design of the experiment, data preprocessing, evaluation of systematic errors, identification of differentially expressed genes, functional classification, and biological interpretation [22], [23]. Kerr and Churchill first established the loop design for microarray experiments [24]. Previous studies demonstrated that loop design is more efficient than reference design because a range of statistical methods can be employed to increase the statistical power and robustness of microarray data analysis [25], [26]. Additionally, the loop-designed approach has a high hybridizations/nodes ratio that markedly increases the empirical power of microarray measurement [27]. In addition, replicating experiments in microarray analysis is important for reliable results. Two kinds of replication are employed for the estimation of variance at different levels: technical and biological replicates. Technical replication is used to estimate system variance such as sample preparation and other effects of artifacts. Biological replication is used to evaluate the variance in biological specimens. Biological variance includes the heterogeneous distribution of cell types and individual variance in genotypes and physiological states. A cautious experimental design should consider both forms of replication, to estimate the variance contributed by the experiment.

DCs are potent antigen-presenting cells that play a prominent role in the development of T cell immune responses [28], [29]. The development of DCs comprises two functional stages. In the immature stage, DCs are localized primarily in peripheral tissues and participate in phagocytosis and the processing of antigens. Following the uptake of antigens, they migrate from the peripheral tissue to the lymphoid organs. During migration, DCs lose the capacity to capture antigens, maturing to become potent antigen presenters to activate T cells [30]. The maturation of DCs is critical to induce antigen-specific T lymphocyte responses and control the differentiation of T cells toward Th1 or Th2 immunity [31], [32]. Our previous results indicated that CS has potential to enhance the immunity of DCs by generating pro-inflammatory cytokines, enhancing the reactivity of allogeneic T cells, and promoting Th1 polarization [17]. On the other hand, CS retards the overactive immunity of LPS on DCs by suppressing the production of LPS-induced pro-inflammatory cytokines, reducing LPS-induced DCs reactivity to allogeneic T cells, and shifting LPS-elicited DC-driven Th1 response towards Th2 [17]. However, the immunomodulatory mechanisms of CS on DCs remain unclear.

The aim of this study was to examine the gene expression profiles of DCs treated with CS, LPS, or LPS/CS using microarray technology. In each set of loop-design microarray experiments, technical replication, performed by identical RNA sampling, in the design of the experiment was employed to estimate systematic variance. For biological replicate, we developed duplicate sets of loop-design microarray experiments to examine two different batches of CS. In addition, in each loop-design microarray experiment, we equally mixed the total RNA of DCs which obtained from three different donors (By pooling the samples of three donors, it reduced the interference of the individual variations. However, we also lost the information of person-to-person variability). We analyzed the common genes found in the two loop-design experiments, which could be used to reduce the variance between different batches of CS. These patterns of gene expression were further analyzed to identify possible pharmacological mechanisms mediated by CS treatment. We incorporated technical and biological replication in the design of the experiments to estimate the systematic and biological variance. This was done to ensure more reliable results. Especially in this study, we are dealing with a biological complex compound, CS, which is not a pure compound.

## Results

### Duplicate Sets of Loop-design Microarray Experiments

**Figure 1:**
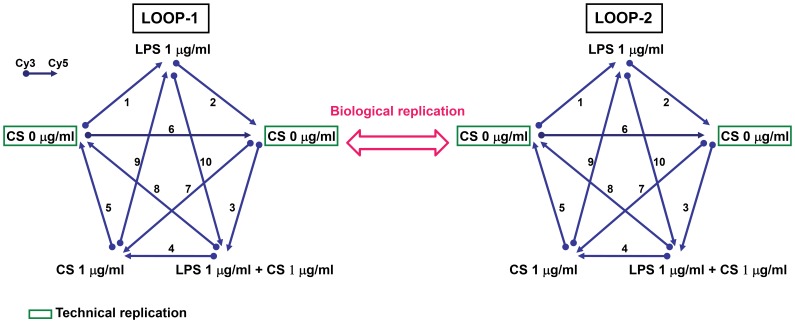
Design of microarray experiment adopting duplicate sets of loop-design microarray experiments. Each experiment contained ten hybridizations and five experimental conditions, two controls and three treatments. Each mRNA sample was a combination of the mRNA of three donors, performed for biological replication in the loop-design microarray experiment. In addition, we utilized duplicate samples in each experiment for technical replication as well as internal control, estimate technological error and enhance the reliability of data.

**Figure 2:**
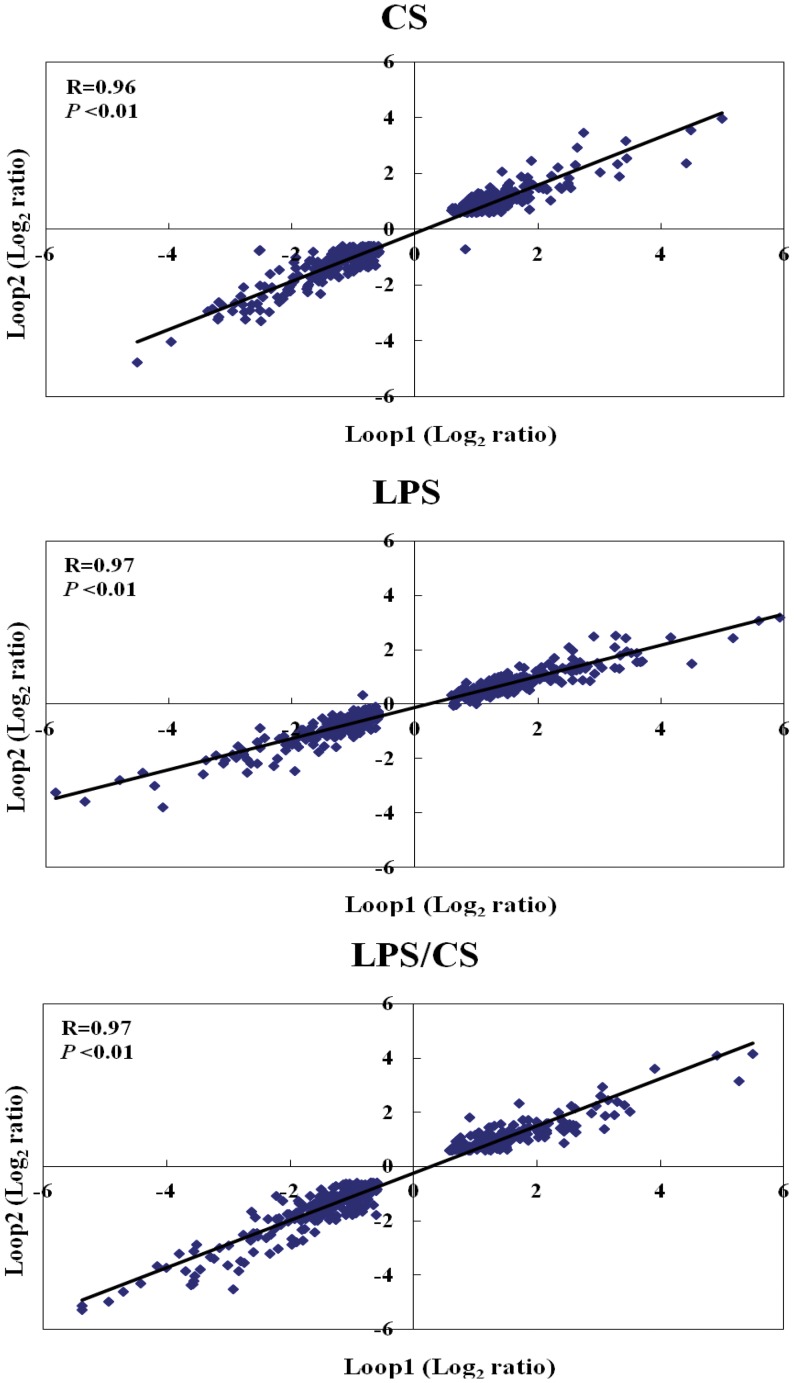
Linear regression of the expression level of common genes found in both sets of loop microarray experiments. The Numbers of common genes found in both loop microarray experiments under treatment with CS, LPS, or LPS/CS are 405, 490, and 453 respectively. Loop1 and loop2 log_2_ ratio denotes the expression ratio between control and treatments (CS, LPS, or LPS/CS) regarding duplicate sets of loop microarray experiments. The correlation coefficients (Rs) in the three lists of common genes are 0.96, 0.97, and 0.97, respectively.

Two types of replication were used in the study: (1) technical replication: identical RNA samples were performed on multiple microarrays as shown in Fig. 1; (2) biological replication: each loop-design microarray experiment was used to assay different batches of CS (Fig. 1). In addition, in each loop-design microarray experiment, we equally mixed the total RNA of DCs which obtained from three different donors to reduce individual variations. In the present study, we utilized duplicate sets of loop-design microarray experiments to examine the effects of two batches of CS. Each loop-design microarray experiment contained ten cDNA microarrays and five experimental conditions, two controls and three treatments (Fig. 1).

### Good Intersection Rate in Two Loop-design Microarray Experiments

Table 1 shows the number of significantly differentially expressed (SDE) genes in two biological replication experiments treated with CS, LPS, or LPS/CS. In experiment loop-1, we found 605, 776, and 725 SDE genes under treatment with CS, LPS, or LPS/CS, respectively (Table 1). In addition, we found 688, 754, and 748 SDE genes under treatment with CS, LPS, or LPS/CS, respectively, in experiment loop-2 (Table 1). The number of common genes between two loop microarray experiments under treatment with CS, LPS, or LPS/CS was 405, 490, and 453, respectively (Table 1). The results indicated that the intersection rate in two loop-design microarray experiments was higher than 60% in CS, LPS, or LPS/CS treatments (Table 1). The expression level of common genes between two loop-design microarray experiments was consistent and the correlation coefficients, Rs were higher than 0.96 (Fig. 2). This high reproducibility of microarray results between the treatments of two different batches of CS, a complex compound, is remarkable. The following analyses are based on the common genes of two loop experiments.

**Table 1:** The number of significantly differentially expressed genes and the intersection rate in two biological replication experiments treated with CS, LPS, and LPS/CS.

Treatment	CS	LPS	LPS/CS
**Genes identified from Loop1 experiment**	605	776	725
**Genes identified from Loop2 experiment**	688	754	748
**Common genes**	405	490	453
**Intersection rate #**	62.6%	64.0%	61.5%

# Intersection rate was calculated by the following equation: common genes/((Loop1 genes+Loop2 genes)/2).

### Assess Genes Regulated by CS, LPS, or LPS/CS Treatment Through Intersection Analysis

**Figure 3:**
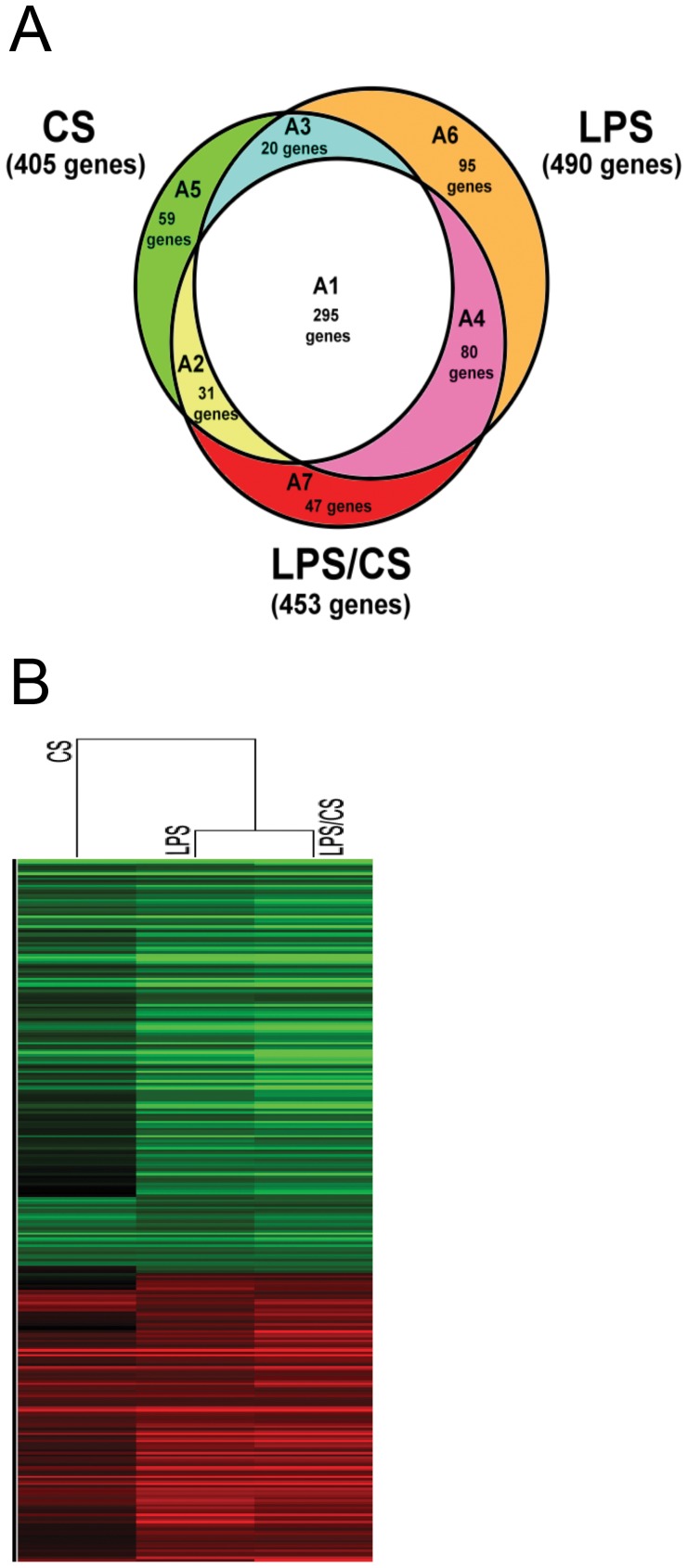
Microarray analysis using intersection analysis and gene clustering. (A) Assesses the genes uniquely regulated by CS, LPS, and LPS/CS through an intersectional method. The SDE genes obtained in both loop experiments were divided into 7 groups. The different regulation genes of A1, A2, A3, A4, A5, A6, and A7 groups were 295, 31, 20, 80, 59, 95, and 47 genes, respectively. (B) The expression of common genes under treatment with CS, LPS, or LPS/CS( A1 group genes) was analyzed by gene clustering (hierarchical model).

As shown in Figure 3A, through the intersection analysis, the genes were divided into 7 groups. The A1 group included the intersecting SDE genes under treatment with CS, LPS, or LPS/CS; A2 group was the intersecting SDE genes under treatment with CS or LPS/CS; A3 group was the intersecting SDE genes under treatment with CS or LPS; A4 group was the intersecting SDE genes under treatment with LPS or LPS/CS (Fig. 3A). The unique SDE genes under treatment with CS, LPS, or LPS/CS were A5, A6, and A7 as shown in Figure 3A, respectively. The numbers SDE genes in A1, A2, A3, A4, A5, A6, and A7 groups were 295, 31, 20, 80, 59, 95, and 47 genes respectively (Fig. 3A).

### Cluster Analysis of Microarray data

For the A1 group genes, we analyzed the correlation of gene expression profiles under treatment with CS, LPS, or LPS/CS by gene clustering. The results showed that LPS treatment had higher correlation with LPS/CS treatment than with CS treatment (Fig. 3B). In addition, we also did a gene clustering analysis of the union genes of these 7 groups. The results showed that LPS treatment also had a higher correlation with LPS/CS treatment than that of CS treatment (Fig. S1). These results indicated that LPS stimulation is a greater contributor to the overall effects of the combined treatment with LPS and CS.

### The Intersecting SDE Genes Under Treatment with CS, LPS, or LPS/CS are Involved in Adjustment of Immune Response and Regulation of Cell Proliferation and Death

We analyzed the A1 group genes by enrichment analysis. The results show that these genes participate mainly in adjustment of immune response and regulation of cell proliferation and death (Table S1). With regard to adjustment of immune response, A1 group genes were mainly involved in the regulation of immune response (13.5%), defense response (11.8%), and inflammatory response (7.6%) (Table S1). The gene expression of A1 group genes under treatment with CS, LPS, or LPS/CS was in accord, despite slight differences (Fig. S2).

### Functional Enrichment Analysis of Uniquely Regulated SDE Genes by CS Treatment

The 59 SDE genes uniquely regulated by CS treatment which we referred to as A5 group genes, were analyzed by functional enrichment analysis. The results show that genes uniquely regulated by CS participated mainly in integral to plasma membrane (22.6%), extracellular region part (15.1%), response to wounding (11.3%), identical protein binding (11.3%), defense response (11.3%), and inflammatory response (9.4 %) (Table 2). In addition, genes uniquely regulated by CS were significantly involved in the regulation of focal adhesion pathway (7.5%), ECM-receptor interaction pathway (5.7%), and hematopoietic cell lineage pathway (5.7%) (Table 2). A detailed list of genes is shown in Table S2.

**Table 2:** Functional enrichment analysis of A5 group genes by GO-terms and KEGG pathway (*P*<0.05).

Term	Category #	Number of genes observed	%	*P* value
integral to plasma membrane	C.C.	12	22.6	1.7×10^−3^
extracellular region part	C.C.	8	15.1	4.3×10^−2^
response to wounding	B.P.	6	11.3	2.4×10^−2^
identical protein binding	M.F.	6	11.3	3.1×10^−2^
defense response	B.P.	6	11.3	4.7×10^−2^
inflammatory response	B.P.	5	9.4	1.7×10^−2^
focal adhesion	KEGG Pathway	4	7.5	4.7×10^−2^
ECM-receptor interaction	KEGG Pathway	3	5.7	4.5×10^−2^
hematopoietic cell lineage	KEGG Pathway	3	5.7	4.7×10^−2^

# Category: B.P. (biological process); C.C. (cellular component); M.F. (molecular function).

### Functional Enrichment Analysis of Uniquely Regulated SDE Genes by LPS Treatment

The 95 SDE genes uniquely regulated by LPS treatment, which we referred to as A6 group genes, were analyzed by functional enrichment analysis. The results show that genes uniquely regulated by LPS participated mainly in immune response, cell proliferation and death, cell signaling, and cell activation (Table S3). In addition, genes uniquely regulated by LPS were significantly involved in regulation of Toll-like receptor signaling pathway (7.5%), systemic lupus erythematosus pathway (6.3%), and complement and coagulation cascades pathway (5.0%) (Table S3).

### Functional Enrichment Analysis of Uniquely Regulated SDE Genes by LPS/CS Treatment

The 47 SDE genes uniquely regulated by LPS/CS treatment, which we referred to as A7 group genes, were analyzed by functional enrichment analysis. The results show that genes uniquely regulated by LPS/CS participated mainly in regulation of enzyme inhibitor activity (10.8%), small GTPase mediated signal transduction (10.8%), cell surface (10.8%), oxidative phosphorylation (8.1%), hydrogen ion transmembrane transporter activity (8.1%), SH3 domain binding (8.1%), aging (8.1%), and antigen processing and presentation of peptide antigens via MHC class I (5.4%) (Table 3). In addition, genes uniquely regulated by LPS/CS were significantly involved in the regulation of oxidative phosphorylation pathway (8.1%) (Table 3). A detailed list of genes is shown in Table S4.

**Table 3:** Functional enrichment analysis of A7 group genes by GO-terms and KEGG pathway (*P*<0.05).

Term	Category #	Number of genes observed	%	*P* value
enzyme inhibitor activity	M.F.	4	10.8	1.9×10^−2^
small GTPase mediated signal transduction	B.P.	4	10.8	2.0×10^−2^
cell surface	C.C.	4	10.8	4.3×10^−2^
oxidative phosphorylation	B.P.	3	8.1	1.4×10^−2^
hydrogen ion transmembrane transporter activity	M.F.	3	8.1	1.6×10^−2^
SH3 domain binding	M.F.	3	8.1	1.7×10^−2^
aging	B.P.	3	8.1	1.8×10^−2^
antigen processing and presentation of peptide antigen via MHC class I	B.P.	2	5.4	4.0×10^−2^
oxidative phosphorylation	KEGG Pathway	3	8.1	3.7×10^−2^

# Category: B.P. (biological process); C.C. (cellular component); M.F. (molecular function).

### Validation of Microarray Data by qRT-PCR

**Figure 4:**
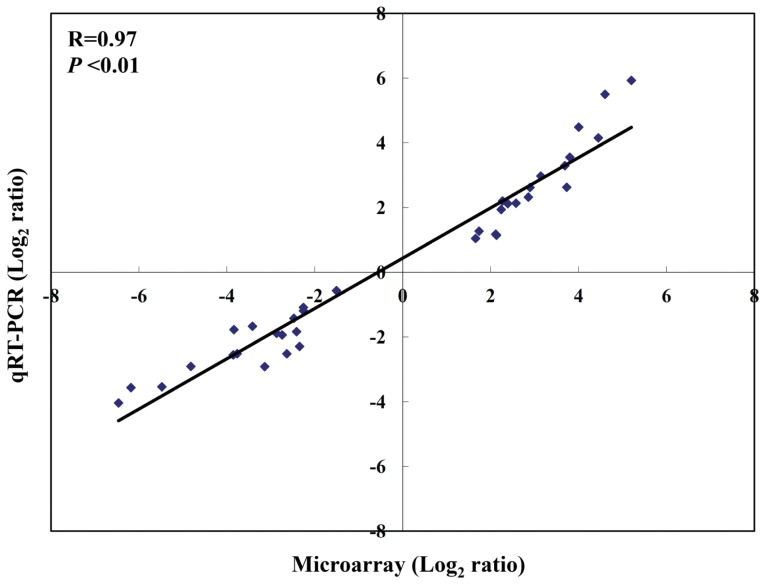
Correlation of gene expression ratios between cDNA microarray and qRT-PCR. A total of 36 microarray samples were validated by qRT-PCR (6 representative genes in different treatments (CS, LPS, and LPS/CS) of duplicate microarrays (Loop1 and Loop2)). Data from both microarray and PCR were normalized by setting the expression level of untreated control.

To further validate the microarray results, qRT-PCR was performed on 6 genes (three up-regulated genes: INDO, CD83, CCL22 and three down-regulated genes: HLA-DRB1, CCL7, CCL18) randomly selected from the A1 group genes using the same RNA samples as were used in the microarray hybridization. A total of 36 microarray samples were validated by qRT-PCR (6 representative genes in three different treatments (CS, LPS, and LPS/CS) of duplicate microarrays loops (Loop1 and Loop2)). Correlation of the expression ratios from the microarray and real-time PCR data are highly correlated (R = 0.97; *P* < 0.01) (Fig. 4).

## Discussion

Chinese herbal medicine is becoming increasingly popular around the world, for the promotion of health and adjuvant therapy [6], [33], [34]. Natural products with multiple pharmacological properties may provide greater clinical effectiveness with fewer side-effects than single compound-based drugs [19]. Our previous study indicated that CS has two side-effects with regard to the regulation of DC activity in different physiology stages, including enhancing the immunity of immature DCs and inhibiting LPS-induced inflammatory response [17]. These two side-effects are a potentially critical issue in balancing the homeostatic steady state of host immunity. In this study, we adopted duplicate sets of loop-design microarray experiments to examine two batches of CS, and attempted to explore the possible mechanism of immunoregulation through treatment with CS.

The loop-design microarray experiments in this study involved technical and biological replication. The technical replication helped us to define the threshold of a 1.5 fold change, reached 1% false discover rate, and was adopted as another selection criterion. To reduce the individual variations, we equally mixed the total RNA of DCs which obtained from three different donors in each loop-design microarray experiment (By pooling the samples of three donors, it reduced the interference of the individual variations. However, we also lost the information of person-to-person variability). The biological replication in this study involved two sets of microarray experiments performed by different individuals using different batches of CS. Through such conscientious controls, our microarray results showed a good intersection rate between two loop-design microarray experiments, with good consistency among the expression of common genes. These results indicate that the data derived from this microarray arrangement is highly accurate and reliable.

Through intersection analysis of microarray results, we identified 205 intersecting SDE genes (A1 group genes), differential expressed under treatment with CS, LPS, or LPS/CS. The gene expression of A1 group genes under treatment with CS, LPS, or LPS/CS was in accord, although it had slight variations. This indicates that the effect caused by CS, LPS, or LPS/CS treatment is very similar. These intersecting SDE genes participate mainly in the adjustment of immune response and the regulation of cell proliferation and death. With regard to the adjustment of immune response, A1 group genes were involved mainly in the regulation of immune response, defense response, and inflammatory response. According to cluster results of A1 group genes, LPS treatment has high correlation with LPS/CS treatment, which indicates that LPS stimulation is a greater contributor to the overall effects of the combined treatment with LPS and CS. In LPS/CS treatment, we treated LPS and CS at the same time. These results revealed that DCs are more responsive in LPS stimulation than that in CS stimulation.

We identified 405 SDE genes under treatment with CS. However, it has 295 genes classified as A1 group genes, which indicate that the effects of CS treatment were similar to LPS treatment. Our previous results indicated that CS could potentially enhance the immunity of DCs by generating pro-inflammatory cytokines, enhancing the reactivity of allogeneic T cells, and promoting the polarization of Th1 [17]. This result showed that the effects of CS treatment resemble those of LPS treatment. We inferred that this may be the reason that so many genes that were differentially regulated by CS treatment were the same as those regulated by LPS. In the results of genes in the A5 group, CS mainly participated in integral to plasma membrane, extracellular region part, response to wounding, identical protein binding, defense response, and inflammatory response. In addition, 59 SDE genes uniquely regulated by CS treatment were significantly involved in the regulation of focal adhesion pathway, ECM-receptor interaction pathway, and hematopoietic cell lineage pathway. The focal adhesion pathway is involved in growth factor signaling, cell proliferation, cell survival and cell migration [35]. The extracellular matrix (ECM) consists of a complex mixture of structural and functional macromolecules and serves an important role in the maintenance of cell and tissue structure and function. These interactions lead to a direct or indirect control of cellular activities such as adhesion, migration, differentiation, proliferation, and apoptosis [36]. These signal transduction pathways contribute to DC adhesion and migration.

We found 95 uniquely regulated SDE genes under treatment with LPS treatment. The genes uniquely regulated by LPS participated mainly in immune response, cell proliferation and death, cell signaling, and cell activation. In addition, they were significantly involved in the regulation of Toll-like receptor signaling pathway, systemic lupus erythematosus pathway, and complement and coagulation cascades pathway. Toll-like receptors signaling pathway, systemic lupus erythematosus pathway, and complement and coagulation cascades pathway are known to be stimulated by LPS [37]–[39].

We identified 453 SDE genes under treatment with LPS/CS. A total of 295 genes were classified as A1 group genes. This indicates that the effects of LPS/CS treatment were similar to those of LPS treatment. According to pathway analysis, 47 SDE genes uniquely regulated by LPS/CS were significantly involved in the regulation of oxidative phosphorylation pathways, including down-regulated expression of *ATP5G3, UCRC,* and *ATP6V1D* (Table S4). We further validated the expression of *ATP5G3, UCRC,* and *ATP6V1D* by qRT-PCR and showed good agreement between real-time RT-PCR and microarray analysis (Figure S3A). Induction of oxidative phosphorylation leads to increased production of reactive oxygen species (ROS) [40]. LPS is known to generate ROS, which is involved in inflammatory processes [41]. Our previous study indicated that CS inhibits LPS-induced inflammatory response [17] may be associated with down-regulation of the oxidative phosphorylation pathway.

A total of 80 SDE genes under treatment with LPS and LPS/CS were classified as A4 group genes. These genes were significantly involved in the adjustment of immune response, regulation of cell proliferation and death, and regulation of tryptophan metabolism pathway (Table S5). We also validated the expression of tryptophan metabolism pathway-associated genes by qRT-PCR and showed good agreement between real-time RT-PCR and microarray analysis (Figure S3B). The immunosuppressive pathway of tryptophan metabolism could lead to the suppression of T cell response and the control of excessive inflammatory reactions [42], [43]. We infer that the inhibition of LPS-induced inflammatory response by CS treatment may be associated with tryptophan metabolic pathway.

In summary, to the best of our knowledge, this work is the first study to use microarray to investigate the effects of CS on the function of the immune system, and assess the effects of CS on DCs at different stages: naïve DCs and LPS-activated DCs. Through intersection analysis of microarray results, we found numerous unique genes under CS, LPS, or LPS/CS treatment and analyzed these genes using functional enrichment analysis. Furthermore, our results identify several pathways mediated by CS on DCs at different stages. These results could provide useful information in further study of the pharmacological mechanisms of CS. This study also demonstrates that with a rigorous experimental design, the biological effects of a complex compound can be reliably studied by a complex system like cDNA microarray.

## Materials and Methods

### Ethics Statement

Fresh whole blood was obtained from normal volunteers at the Taiwan Blood Center by an Institutional Review Board (IRB) approved procedure issued by National Tsing Hua University, Hsinchu, Taiwan.

### Reagents

The culture medium was RPMI 1640 (Gibco-BRL, Life Technologies, Paisley, UK) supplemented with 2 mM L-glutamine, 25 mM HEPES, 100 U/ml penicillin, 0.1 mg/ml streptomycin (Gibco-BRL, Life Technologies, Paisley, UK), and 10% heat-inactivated FCS (Hyclone, Logan, UT, USA). Recombinant human GM-CSF and recombinant human IL-4 were purchased from PeproTech (Rocky Hill, NJ, USA). LPS (Escherichia coli serotype O55: B5) was purchased from Sigma (St. Louis, MO, USA). Corning UltraGAPS slides were purchased from Corning Incorporated (Acton, MA, USA). The 3DNA array 900 labeling kit was purchased from Genisphere (Hatfield, PA, USA). The RNeasy mini kit was purchased from Qiagen (Valencia, CA, USA). Human cDNA microarray probe was purchased from Incyte Genomics (Palo Alto, CA, USA) The SuperScript® II was purchased from Gibco-Invitrogen (Carlsbad, CA, USA). The SpotReport™ cDNA Array Validation System was purchased from Stratagene (La Jolla, CA, USA). The Agilent 2100 bioanalyzer and RNA 6000 Nano LabChip kit were purchased from Agilent Technologies ( PaloAlto, CA, USA).

### Preparation of Hot-water Extracts of CS

CS was purchased from the Chinese Medicine Drug store, Taipei, Taiwan. To guarantee the quality of the CS, the genetic variation was analyzed by the DNA sequencing as previously described [17]. Experimental results confirmed that CS samples used in this study are genuine CS, and the results were submitted to the European Molecular Biology Laboratory database. The accession numbers of the five CS samples are FM164741, FM164742, FM164743, FM164744, and FM164745 [17]. Different sample batches of Chinese herbal medicine may have different levels of active ingredients. To reduce the variance of different CS batches, we used two batches of CS in this study. The CS extracted with hot water was obtained as previously described [33]. Briefly, CS samples were dried at 45°C in the dark to a constant weight and pulverized. Two grams of the CS sample was dissolved in 40 ml water and hot-water extraction was performed at 90°C for 2 h. After centrifugation at 3,000 g for 20 min, the supernatant was harvested and sterilized by filtration through a 0.22 µm filter and stored at −20°C until used. The pH value of CS extracts was measured by Corning pH 320 meter (Corning, NY, USA) and the measured value is 7.49 ± 0.02. To examine potential endotoxin contamination, CS extracts were measured by LAL assay. Results indicated the two batches of CS had undetectable levels (<0.05 endotoxin units/ml) of LPS (data not shown). Moreover, as indicated in our previous study that CS extracts were chemically fingerprinted by HPLC, the results demonstrated that the major components of CS are extracted [17].

### Generation of Human Monocyte-derived DCs

Human peripheral blood mononuclear cells (PBMCs) were isolated by Ficoll-Hypaque density gradient centrifugation. Monocytes were purified following the plastic adherence method [44]. A total of 10^7^ cells/well in 6-well flat-bottom plates were incubated in RPMI 1640 culture medium. After 2 h incubation at 37°C in humidified air containing 5 % CO_2_, nonadherent cells were removed by gentle washing and plastic-adherent cells were used as monocytes. This monocyte population exhibited >90% CD14 positive staining, as revealed by flow cytometric analysis (data not shown). DCs were generated from monocytes that were cultured at 37°C in an incubator with 5% humidified CO_2_ in RPMI 1640 culture medium that was supplemented with recombinant human GM-CSF 500 U/ml and recombinant human IL-4 1000 U/ml for 6 days. On days 2 and 4, half of the medium was replaced with fresh medium containing recombinant human GM-CSF and recombinant human IL-4. On day 6, immature DCs were reseeded into a 6-well culture plate at a total of 10^6^ cells/well and treated with various concentrations of CS extracts (0 and 1 µg/ml) in the absence or presence of LPS (1 µg/ml) in a culture for 2 days. The viability of the cells under these treatments exceeded 90% (data not shown), based on results of MTT assay that were performed following the manufacturer’s instructions (Sigma, St. Louis, MO, USA).

### Microarray Fabrication

A total of 7,334 sequence-verified human cDNA clones, ten Arabidopsis cDNAs (SpotReport™ cDNA Array Validation System) to serve as spike-in controls, and one housekeeping gene (β-actin) to serve as a positive control, were arrayed on Corning UltraGAPS slides. Quadruplicate spotting of 7,334 human cDNA and the 96 spottings of Arabidopsis cDNA and housekeeping genes were performed on every array, to enhance the statistical confidence in the gene expression data. Each array had 32,448 spots. The arrays were post-processed, based on the Corning Instruction Manual for UltraGAPS Coated Slides.

### RNA Extraction and Microarray Hybridization

DCs were harvested at scheduled sampling times to extract total RNA using an RNeasy mini kit following manufacturer’s protocol (Qiagen, Valencia, CA, USA). In addition, we mixed the RNA of three different donors’ in each loop-design microarray experiment. The quality of total RNA was evaluated using the Agilent 2100 bioanalyzer with the RNA 6000 Nano LabChip kit. Before reverse transcription, each sample RNA was spiked with a mixture of Arabidopsis mRNAs. Fluorescence-labeled cDNA was conducted using 3DNA Array 900 labeling kit, following the manufacturer’s protocols (Genisphere, Hatfield, PA, USA). Reverse transcription was performed using SuperScript II (Invitrogen, Carlsbad, CA, USA). Hybridization was performed at 65°C in a water bath for 16 to 18 hours, and arrays were washed following the manufacturer’s protocol (Corning Life Sciences, New York, NY, USA). The arrays were scanned using the GenePix 4000B scanner (Axon Instruments, Foster City, CA, USA).

### Microarray Data Analysis and Statistical Analysis

Microarray data preprocessing, normalization, and statistical analysis was performed using a bioinformatics software suite called Tsing Hua Engine for Microarray Experiment (THEME) [45]. Spot-screening rules were applied to screen out invalid spots on arrays. The spot-screening rules were as follows. (i) Exclude spots defined as “flag bad” or “absent” in all GPR files; (ii) exclude spots with a diameters of less than 75 µm; (iii) exclude spots with a coefficient of variation (CV) of pixel intensity of over 100% in both channels; (iv) exclude spots whose signal to noise ratios (SNRs) in both channels was less than 2 in loop-1 experiments and less than 3 in loop-2 experiments. The signal to noise ratio is defined as (S-B)/B (“S”: mean of pixel intensities of “signal”; “B”: median pixel intensity of “background”).

The logarithm of the ratios for all valid spots on each array was normalized by pin-wise normalization [46]. After data preprocessing, the normalized log ratios of the cDNAs were processed using a log linear model, which was described in our previous study [21]. 5550 and 5843 genes satisfied the selection criteria in the two biological replication loop experiments. F test was used to identify differentially expressed genes. Differentially regulated cDNA clones were identified by applying Bonferroni-adjusted *P* < 0.05 for each null hypothesis in combination with at least a 1.5-fold change [21]. According to the technical replication experiments, a 1.5-fold change, reached 1% false discover rate, and was adopted as another selection criterion. The microarray data is available at GEO (GSE24191).

### Gene Clustering, Functional Annotation and Functional Enrichment Analysis

Gene clustering was performed by Gene cluster 3.0 and TreeView software [47]. The functional annotation of differentially regulated genes was analyzed using DAVID bioinformatics resources based on the biological process category at level 3. Functional enrichment analysis was performed using the online Database for Annotation, Visualization and Integrated Discovery (DAVID), which is available at http://david.abcc.ncifcrf.gov/ [48], [49]. Differentially regulated gene lists identified at each time point were compared with a list of 7,334 clones to identify significantly overrepresented KEGG pathways and GO terms. KEGG pathways or GO terms containing more than two genes and a Fisher Exact *p*-value of less than 0.05 were regarded as significant.

### Quantitative RT-PCR (qRT-PCR)

We confirmed selected microarray results by comparison with mRNA levels obtained by q*RT-PCR* using selected gene-specific primer pairs which were designed using Primer Express 3.0 (Applied Biosystems) (Table S6). Total RNA was isolated using an RNeasy mini kit following manufacturer’s protocol (Qiagen, Valencia, CA, USA). cDNA was synthesized from 1 to 2 g RNA using oligo-dT (Roche, Indianapolis, IN, USA) and SuperScript II reverse transcriptase kit (Invitrogen, Carlsbad, CA, USA) according to manufacturer’s instruction. The PCR reaction was performed in triplicates using ABI 7300 real-time PCR system (Applied Biosystems, Foster City, CA, USA) with QuantiTect SYBR green RT-PCR kit (Qiagen, Germany). All quantitations (threshold cycle [CT] values) were normalized to that of GAPDH to generate ΔCT, and the difference among the ΔCT value of the sample and that of the reference (uninfected sample) was calculated as -ΔΔCT. The relative level of gene expression was expressed as 2-ΔΔCT.

## Supporting Information

Figure S1 **Analysis of the expression level of union genes treated with CS, LPS, or LPS/CS through hierarchical clustering.** The expression level of union genes treated with CS, LPS, or LPS/CS as a combination of A1, A2, A3, A4, A5, A6, and A7 group genes by gene clustering (hierarchical model).
(TIF)

Figure S2 **Expression level of A1 group genes treated with CS, LPS, or LPS/CS.**
(TIF)

Figure S3 **Validation of microarray data by qRT-PCR.** (A) Three genes significantly involved in the regulation of oxidative phosphorylation pathways under LPS/CS treatment as A7 group genes were validated by qRT-PCR. (B) Four genes significantly involved in the regulation of tryptophan metabolism pathway under both LPS (A6 group genes) and LPS/CS treatment (A7 group genes) were validated by qRT-PCR.
(TIF)

Table S1 **Functional enrichment analysis of A1 group genes by GO-terms (** ***P*** **< 0.01).**
(DOC)

Table S2 **Gene list of A5 group genes analyzed by functional enrichment analysis (** ***P*** **< 0.05).**
(DOC)

Table S3 **Functional enrichment analysis of A6 group genes by GO-terms and KEGG pathway (** ***P*** **< 0.01).**
(DOC)

Table S4 **Gene list of A7 group genes analyzed by functional enrichment analysis (** ***P*** **< 0.05).**
(DOC)

Table S5 **Functional enrichment analysis of A4 group genes by GO-terms and KEGG pathway (** ***P*** **< 0.05).**
(DOC)

Table S6 **Primers for real-time PCR.**
(DOC)
