# Supplementary figures and images for: Gene Expression Profiling of Dendritic Cells in Different Physiological Stages under Cordyceps sinensis Treatment
Source: PLoS One. 2012 Jul 19;7(7):e40824. doi: 10.1371/journal.pone.0040824 (PMC3400664; doi:10.1371/journal.pone.0040824)

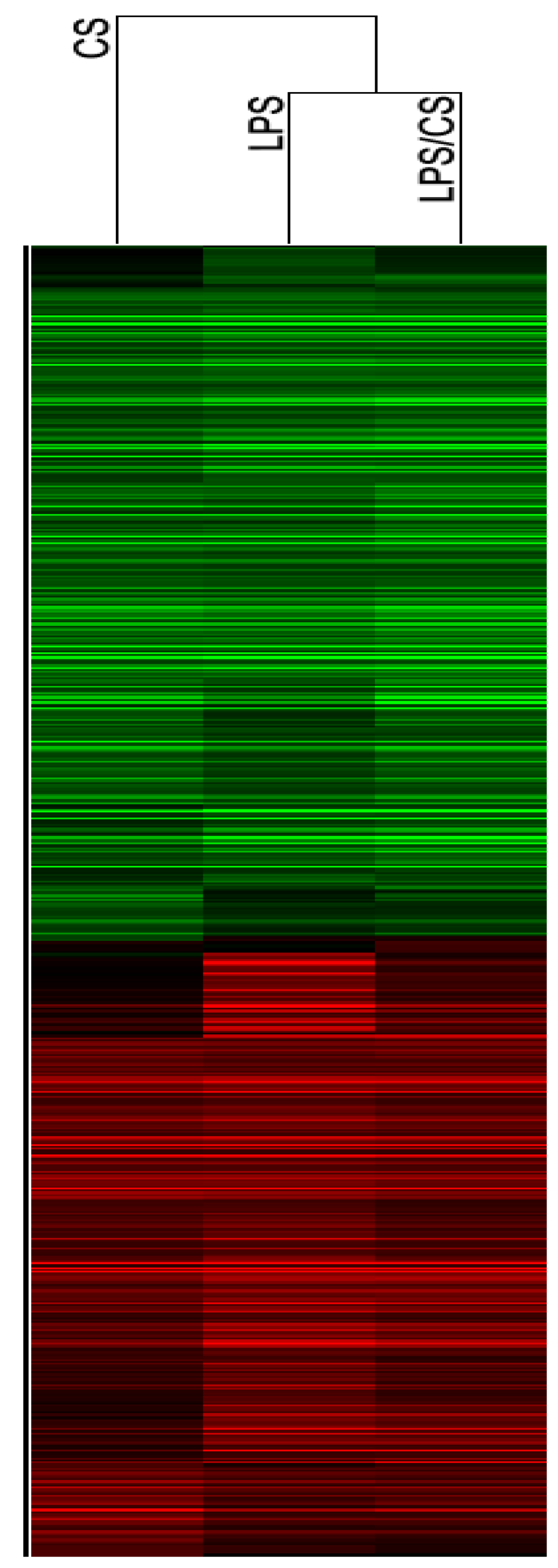

Supplement: Figure S1 — Analysis of the expression level of union genes treated with CS, LPS, or LPS/CS through hierarchical clustering. The expression level of union genes treated with CS, LPS, or LPS/CS as a combination of A1, A2, A3, A4, A5, A6, and A7 group genes by gene clustering (hierarchical model). (TIF) [file pone.0040824.s001.tif]

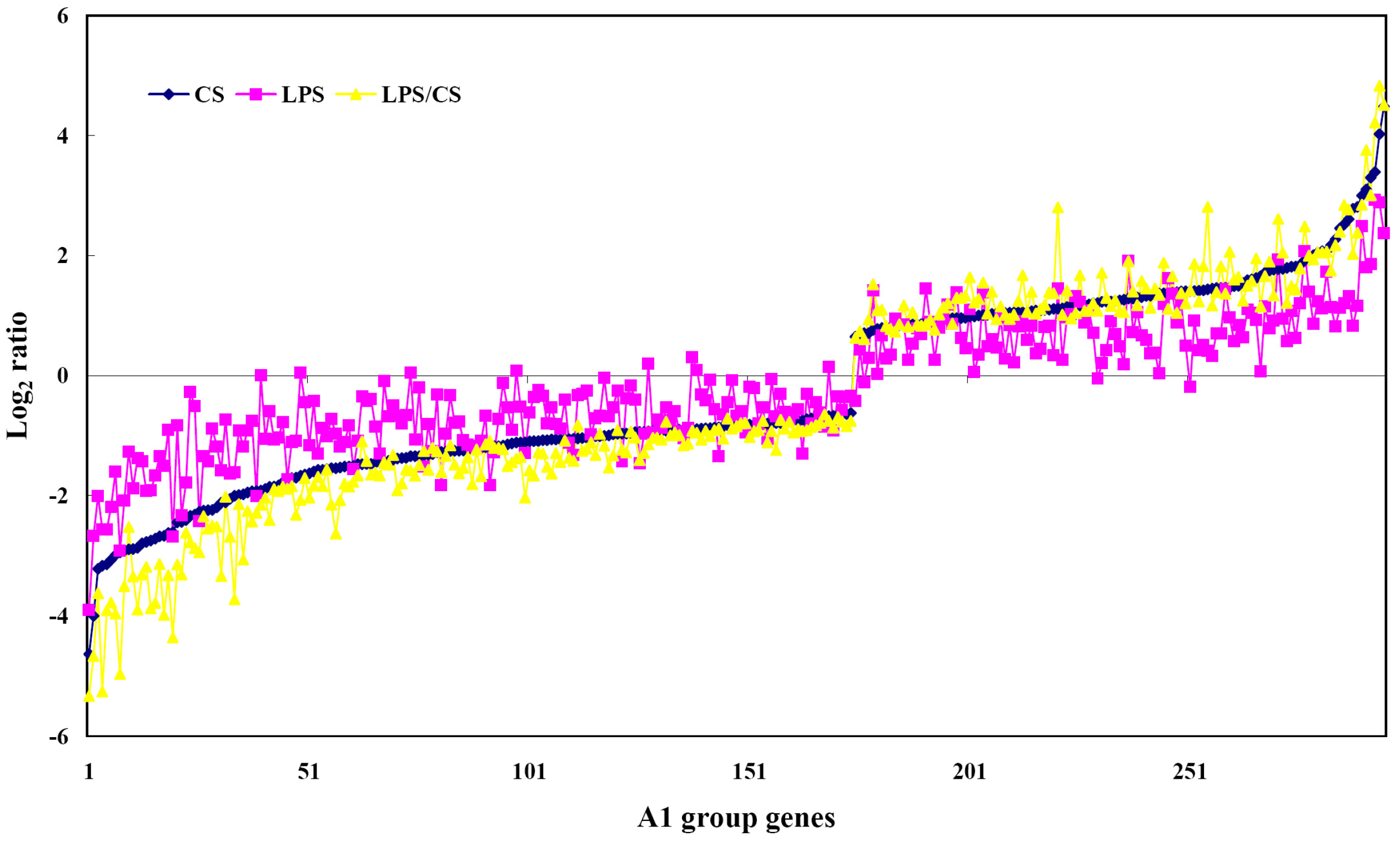

Supplement: Figure S2 — Expression level of A1 group genes treated with CS, LPS, or LPS/CS. (TIF) [file pone.0040824.s002.tif]

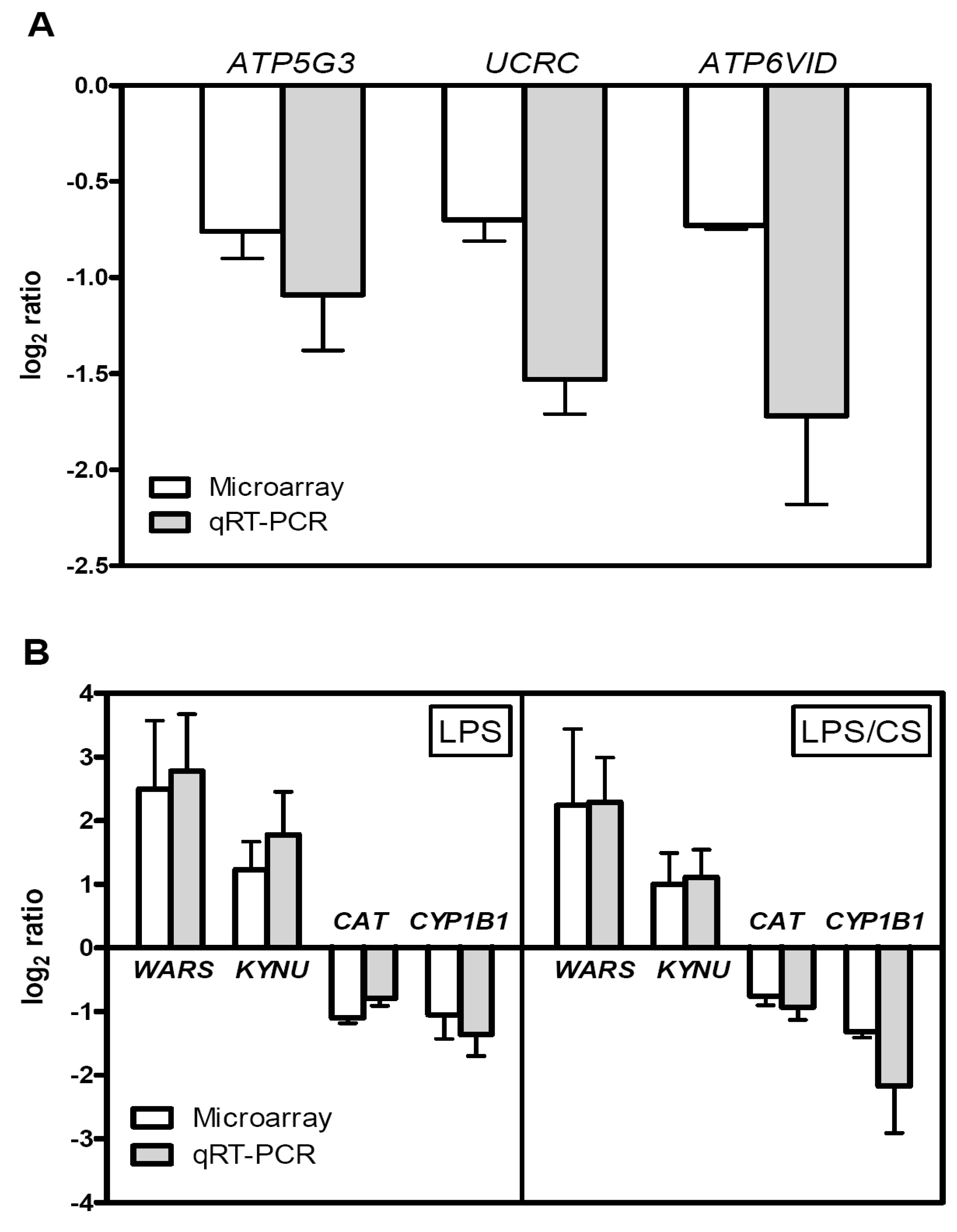

Supplement: Figure S3 — Validation of microarray data by qRT-PCR. (A) Three genes significantly involved in the regulation of oxidative phosphorylation pathways under LPS/CS treatment as A7 group genes were validated by qRT-PCR. (B) Four genes significantly involved in the regulation of tryptophan metabolism pathway under both LPS (A6 group genes) and LPS/CS treatment (A7 group genes) were validated by qRT-PCR. (TIF) [file pone.0040824.s003.tif]
