# Supplementary material for: Gene Expression Profiling of Dendritic Cells in Different Physiological Stages under Cordyceps sinensis Treatment
Source: PLoS One. 2012 Jul 19;7(7):e40824. doi: 10.1371/journal.pone.0040824 (PMC3400664; doi:10.1371/journal.pone.0040824)
Supplement: Table S1 — Functional enrichment analysis of A1 group genes by GO-terms ( P < 0.01). (DOC) [file pone.0040824.s004.doc]

| **Table S1.** Functional enrichment analysis of A1 group genes by GO-terms (*P*< 0.01). | | | | |  |  |  |  |
| --- | --- | --- | --- | --- | --- | --- | --- | --- |
| **Term** | | | | | **Category #** | **Number of genes observed** | **%** | ***P* value** |
| ***Immune response*** | | | | |  |  |  |  |
| immune response | | | | | B.P. | 32 | 13.5 | 2.7 x 10^-8^ |
| defense response | | | | | B.P. | 28 | 11.8 | 4.7 x 10^-7^ |
| innate immune response | | | | | B.P. | 8 | 3.4 | 4.4 x 10^-3^ |
| positive regulation of inflammatory response | | | | | B.P. | 4 | 1.7 | 9.5 x 10^-3^ |
| antigen processing and presentation of peptide or polysaccharide antigen via MHC class II | | | | | B.P. | 4 | 1.7 | 8.7 x 10^-3^ |
| MHC class II protein complex | | | | | C.C. | 4 | 1.7 | 7.0 x 10^-3^ |
| regulation of interleukin-12 production | | | | | B.P. | 4 | 1.7 | 1.9 x 10^-3^ |
| natural killer cell mediated cytotoxicity | | | | | B.P. | 3 | 1.3 | 6.8 x 10^-3^ |
| natural killer cell mediated immunity | | | | | B.P. | 3 | 1.3 | 6.8 x 10^-3^ |
| regulation of interleukin-12 biosynthetic process | | | | | B.P. | 3 | 1.3 | 6.8 x 10^-3^ |
| IgE binding | | | | | M.F. | 3 | 1.3 | 1.9 x 10^-3^ |
| regulation of immune effector process | | | | | B.P. | 8 | 3.4 | 6.3 x 10^-4^ |
| regulation of lymphocyte activation | | | | | B.P. | 9 | 3.8 | 1.5 x 10^-3^ |
| positive regulation of immune response | | | | | B.P. | 9 | 3.8 | 1.2 x 10^-3^ |
| inflammatory response | | | | | B.P. | 18 | 7.6 | 4.2 x 10^-6^ |
| positive regulation of immune system process | | | | | B.P. | 14 | 5.9 | 4.2 x 10^-5^ |
| regulation of cytokine production | | | | | B.P. | 11 | 4.6 | 2.8 x 10^-4^ |
| regulation of leukocyte activation | | | | | B.P. | 11 | 4.6 | 1.6 x 10^-4^ |
| antigen processing and presentation | | | | | B.P. | 7 | 3.0 | 1.2 x 10^-3^ |
| regulation of B cell activation | | | | | B.P. | 5 | 2.1 | 6.3 x 10^-3^ |
| cytokine production | | | | | B.P. | 5 | 2.1 | 4.7 x 10^-3^ |
| lymphocyte mediated immunity | | | | | B.P. | 6 | 2.5 | 5.3 x 10^-3^ |
| positive regulation of cytokine biosynthetic process | | | | | B.P. | 6 | 2.5 | 6.4 x 10^-4^ |
| response to virus | | | | | B.P. | 7 | 3.0 | 4.4 x 10^-3^ |
|  | | | | |  |  |  |  |
| ***Cell proliferation/deadth*** | | | | |  |  |  |  |
| regulation of cell death | | | | | B.P. | 39 | 16.5 | 1.3 x 10^-10^ |
| regulation of programmed cell death | | | | | B.P. | 39 | 16.5 | 1.2 x 10^-10^ |
| regulation of apoptosis | | | | | B.P. | 39 | 16.5 | 9.3 x 10^-11^ |
| regulation of cell proliferation | | | | | B.P. | 25 | 10.5 | 5.2 x 10^-4^ |
| death | | | | | B.P. | 20 | 8.4 | 9.6 x 10^-3^ |
| cell death | | | | | B.P. | 20 | 8.4 | 9.0 x 10^-3^ |
| positive regulation of cell death | | | | | B.P. | 20 | 8.4 | 1.9 x 10^-5^ |
| positive regulation of programmed cell death | | | | | B.P. | 20 | 8.4 | 1.8 x 10^-5^ |
| positive regulation of apoptosis | | | | | B.P. | 20 | 8.4 | 1.6 x 10^-5^ |
| negative regulation of cell death | | | | | B.P. | 19 | 8.0 | 5.9 x 10^-6^ |
| negative regulation of programmed cell death | | | | | B.P. | 19 | 8.0 | 5.7 x 10^-6^ |
| negative regulation of apoptosis | | | | | B.P. | 19 | 8.0 | 4.7 x 10^-6^ |
| induction of programmed cell death | | | | | B.P. | 16 | 6.8 | 7.9 x 10^-5^ |
| induction of apoptosis | | | | | B.P. | 16 | 6.8 | 7.6 x 10^-5^ |
| apoptotic mitochondrial changes | | | | | B.P. | 4 | 1.7 | 9.5 x 10^-3^ |
| anti-apoptosis | | | | | B.P. | 12 | 5.1 | 2.1 x 10^-4^ |
|  | | | | |  |  |  |  |
| ***Others*** | | | | |  |  |  |  |
| plasma membrane part | | | | | C.C. | 52 | 21.9 | 6.2 x 10^-4^ |
| extracellular region part | | | | | C.C. | 27 | 11.4 | 2.2 x 10^-3^ |
| response to wounding | | | | | B.P. | 26 | 11.0 | 1.8 x 10^-7^ |
| extracellular space | | | | | C.C. | 24 | 10.1 | 2.3 x 10^-4^ |
| response to organic substance | | | | | B.P. | 23 | 9.7 | 9.5 x 10^-4^ |
| vacuole | | | | | C.C. | 14 | 5.9 | 1.2 x 10^-4^ |
| lytic vacuole | | | | | C.C. | 14 | 5.9 | 2.0 x 10^-5^ |
| lysosome | | | | | C.C. | 14 | 5.9 | 2.0 x 10^-5^ |
| protein homodimerization activity | | | | | M.F. | 12 | 5.1 | 8.6 x 10^-3^ |
| positive regulation of response to stimulus | | | | | B.P. | 12 | 5.1 | 6.9 x 10^-4^ |
| regulation of cell activation | | | | | B.P. | 12 | 5.1 | 5.2 x 10^-5^ |
| protein complex binding | | | | | M.F. | 9 | 3.8 | 7.2 x 10^-3^ |
| wound healing | | | | | B.P. | 9 | 3.8 | 7.1 x 10^-3^ |
| external side of plasma membrane | | | | | C.C. | 9 | 3.8 | 4.3 x 10^-3^ |
| positive regulation of cell activation | | | | | B.P. | 8 | 3.4 | 1.2 x 10^-3^ |
| regulation of cytokine biosynthetic process | | | | | B.P. | 8 | 3.4 | 1.1 x 10^-4^ |
| positive regulation of leukocyte activation | | | | | B.P. | 7 | 3.0 | 4.6 x 10^-3^ |
| membrane lipid metabolic process | | | | | B.P. | 6 | 2.5 | 5.6 x 10^-3^ |
| response to hydrogen peroxide | | | | | B.P. | 5 | 2.1 | 9.9 x 10^-3^ |
| integrin binding | | | | | M.F. | 5 | 2.1 | 9.9 x 10^-3^ |
| cell killing | | | | | B.P. | 5 | 2.1 | 4.2 x 10^-4^ |
| # Category: B.P. (biological process); C.C. (cellular componet); M.F. (molecular function). | | | | |  |  |  |  |
|  |  |  |  |  | | | | |
