# Supplementary material for: Gene Expression Profiling of Dendritic Cells in Different Physiological Stages under Cordyceps sinensis Treatment
Source: PLoS One. 2012 Jul 19;7(7):e40824. doi: 10.1371/journal.pone.0040824 (PMC3400664; doi:10.1371/journal.pone.0040824)
Supplement: Table S2 — Gene list of A5 group genes analyzed by functional enrichment analysis ( P < 0.05). (DOC) [file pone.0040824.s005.doc]

| **Table S2.** Gene list of A5 group genes analyzed by functional enrichment analysis (*P*< 0.05). | | | | | | |
| --- | --- | --- | --- | --- | --- | --- |
| **Gene description** | **Gene name** | **Entrez ID** | **Loop1 (log2 ratio)** | **Loop2 (log2 ratio)** | **Mean** | **SD** |
| ***integral to plasma membrane*** |  |  |  |  |  |  |
| CDw44 antigen | CD44 | 960 | -1.28 | -1.04 | -1.16 | 0.17 |
| sulfate transporter (DTD) | SLC26A2 | 1836 | 1.20 | 0.61 | 0.90 | 0.42 |
| ecotropic viral integration site 2B | EVI2B | 2124 | -0.71 | -0.81 | -0.76 | 0.07 |
| lymphocyte IgE receptor (low affinity receptor Fc epsilon R) | FCER2 | 2208 | -1.79 | -1.37 | -1.58 | 0.29 |
| interleukin 13 receptor, alpha 1 | IL13RA1 | 3597 | 0.73 | 0.68 | 0.70 | 0.03 |
| integrin, alpha 4 (antigen CD49D, alpha 4 subunit of VLA-4 receptor) | ITGA4 | 3676 | 0.62 | 0.76 | 0.69 | 0.10 |
| integrin, beta 7 | ITGB7 | 3695 | 1.32 | 1.16 | 1.24 | 0.11 |
| sodium channel, voltage-gated, type IX, alpha subunit | SCN9A | 6335 | -1.65 | -0.81 | -1.23 | 0.59 |
| retroviral receptor mRNA | SLC7A1 | 6541 | -0.74 | -0.90 | -0.82 | 0.12 |
| testis enhanced gene transcript protein (TEGT) | TMBIM6 | 7009 | -0.60 | -0.65 | -0.63 | 0.03 |
| thrombomodulin | THBD | 7056 | -1.27 | -1.03 | -1.15 | 0.17 |
| IFN-alpha2b-inducing related protein 1 (IFNRG1) | CLEC2B | 9976 | -0.66 | -0.91 | -0.79 | 0.17 |
|  |  |  |  |  |  |  |
| ***extracellular region part*** |  |  |  |  |  |  |
| CDw44 antigen | CD44 | 960 | -1.28 | -1.04 | -1.16 | 0.17 |
| EGF-containing fibulin-like extracellular matrix protein 1 | EFEMP1 | 2202 | 0.80 | 0.62 | 0.71 | 0.13 |
| insulin-degrading enzyme | IDE | 3416 | 1.02 | 0.60 | 0.81 | 0.29 |
| interleukin 1 receptor antagonist | IL1RN | 3557 | -1.01 | -0.66 | -0.84 | 0.25 |
| lectin, galactoside-binding, soluble, 1 | LGALS1 | 3956 | 0.94 | 0.91 | 0.93 | 0.02 |
| thrombomodulin | THBD | 7056 | -1.27 | -1.03 | -1.15 | 0.17 |
| chemokine (C-C motif) ligand 4-like 1; chemokine (C-C motif) ligand 4-like 2 | CCL4L1 | 9560 | -0.60 | -0.85 | -0.72 | 0.18 |
| granulysin | GNLY | 10578 | 0.83 | -0.72 | 0.05 | 1.09 |
|  |  |  |  |  |  |  |
| ***response to wounding*** |  |  |  |  |  |  |
| CDw44 antigen | CD44 | 960 | -1.28 | -1.04 | -1.16 | 0.17 |
| CCAAT/enhancer binding protein (C/EBP), beta | CEBPB | 1051 | -0.72 | -0.73 | -0.72 | 0.01 |
| interleukin 1 receptor antagonist | IL1RN | 3557 | -1.01 | -0.66 | -0.84 | 0.25 |
| sodium channel, voltage-gated, type IX, alpha subunit | SCN9A | 6335 | -1.65 | -0.81 | -1.23 | 0.59 |
| thrombomodulin | THBD | 7056 | -1.27 | -1.03 | -1.15 | 0.17 |
| chemokine (C-C motif) ligand 4-like 1; chemokine (C-C motif) ligand 4-like 2 | CCL4L1 | 9560 | -0.60 | -0.85 | -0.72 | 0.18 |
|  |  |  |  |  |  |  |
| ***identical protein binding*** |  |  |  |  |  |  |
| CCAAT/enhancer binding protein (C/EBP), beta | CEBPB | 1051 | -0.72 | -0.73 | -0.72 | 0.01 |
| filamin A, alpha (actin binding protein 280) | FLNA | 2316 | -0.77 | -0.74 | -0.76 | 0.02 |
| insulin-degrading enzyme | IDE | 3416 | 1.02 | 0.60 | 0.81 | 0.29 |
| integrin, alpha 4 (antigen CD49D, alpha 4 subunit of VLA-4 receptor) | ITGA4 | 3676 | 0.62 | 0.76 | 0.69 | 0.10 |
| integrin, beta 7 | ITGB7 | 3695 | 1.32 | 1.16 | 1.24 | 0.11 |
| lectin, galactoside-binding, soluble, 1 | LGALS1 | 3956 | 0.94 | 0.91 | 0.93 | 0.02 |
|  |  |  |  |  |  |  |
| ***defense response*** |  |  |  |  |  |  |
| CDw44 antigen | CD44 | 960 | -1.28 | -1.04 | -1.16 | 0.17 |
| CCAAT/enhancer binding protein (C/EBP), beta | CEBPB | 1051 | -0.72 | -0.73 | -0.72 | 0.01 |
| interleukin 1 receptor antagonist | IL1RN | 3557 | -1.01 | -0.66 | -0.84 | 0.25 |
| sodium channel, voltage-gated, type IX, alpha subunit | SCN9A | 6335 | -1.65 | -0.81 | -1.23 | 0.59 |
| chemokine (C-C motif) ligand 4-like 1; chemokine (C-C motif) ligand 4-like 2 | CCL4L1 | 9560 | -0.60 | -0.85 | -0.72 | 0.18 |
| granulysin | GNLY | 10578 | 0.83 | -0.72 | 0.05 | 1.09 |
|  |  |  |  |  |  |  |
| ***inflammatory response*** |  |  |  |  |  |  |
| CDw44 antigen | CD44 | 960 | -1.28 | -1.04 | -1.16 | 0.17 |
| CCAAT/enhancer binding protein (C/EBP), beta | CEBPB | 1051 | -0.72 | -0.73 | -0.72 | 0.01 |
| interleukin 1 receptor antagonist | IL1RN | 3557 | -1.01 | -0.66 | -0.84 | 0.25 |
| sodium channel, voltage-gated, type IX, alpha subunit | SCN9A | 6335 | -1.65 | -0.81 | -1.23 | 0.59 |
| chemokine (C-C motif) ligand 4-like 1; chemokine (C-C motif) ligand 4-like 2 | CCL4L1 | 9560 | -0.60 | -0.85 | -0.72 | 0.18 |
|  |  |  |  |  |  |  |
| ***focal adhesion pathway*** |  |  |  |  |  |  |
| cyclin D3 | CCND3 | 896 | -0.80 | -0.63 | -0.72 | 0.12 |
| filamin A, alpha (actin binding protein 280) | FLNA | 2316 | -0.77 | -0.74 | -0.76 | 0.02 |
| integrin, alpha 4 (antigen CD49D, alpha 4 subunit of VLA-4 receptor) | ITGA4 | 3676 | 0.62 | 0.76 | 0.69 | 0.10 |
| integrin, beta 7 | ITGB7 | 3695 | 1.32 | 1.16 | 1.24 | 0.11 |
|  |  |  |  |  |  |  |
| ***ECM-receptor interaction pathway*** |  |  |  |  |  |  |
| CDw44 antigen | CD44 | 960 | -1.28 | -1.04 | -1.16 | 0.17 |
| integrin, alpha 4 (antigen CD49D, alpha 4 subunit of VLA-4 receptor) | ITGA4 | 3676 | 0.62 | 0.76 | 0.69 | 0.10 |
| integrin, beta 7 | ITGB7 | 3695 | 1.32 | 1.16 | 1.24 | 0.11 |
|  |  |  |  |  |  |  |
| ***hematopoietic cell lineage pathway*** |  |  |  |  |  |  |
| CDw44 antigen | CD44 | 960 | -1.28 | -1.04 | -1.16 | 0.17 |
| lymphocyte IgE receptor (low affinity receptor Fc epsilon R) | FCER2 | 2208 | -1.79 | -1.37 | -1.58 | 0.29 |
| integrin, alpha 4 (antigen CD49D, alpha 4 subunit of VLA-4 receptor) | ITGA4 | 3676 | 0.62 | 0.76 | 0.69 | 0.10 |
