# Supplementary material for: Gene Expression Profiling of Dendritic Cells in Different Physiological Stages under Cordyceps sinensis Treatment
Source: PLoS One. 2012 Jul 19;7(7):e40824. doi: 10.1371/journal.pone.0040824 (PMC3400664; doi:10.1371/journal.pone.0040824)
Supplement: Table S3 — Functional enrichment analysis of A6 group genes by GO-terms and KEGG pathway ( P < 0.01). (DOC) [file pone.0040824.s006.doc]

| **Table S3.** Functional enrichment analysis of A6 group genes by GO-terms and KEGG pathway (*P*< 0.01). | | | | |
| --- | --- | --- | --- | --- |
| **Term** | **Category #** | **Number of genes observed** | **%** | ***P* value** |
| ***Immune response*** |  |  |  |  |
| defense response | B.P | 17 | 21.3 | 1.8 x 10^-8^ |
| immune response | B.P | 14 | 17.5 | 1.5 x 10^-5^ |
| inflammatory response | B.P | 13 | 16.3 | 2.0 x 10^-8^ |
| positive regulation of immune system process | B.P | 9 | 11.3 | 1.2 x 10^-5^ |
| cytokine activity | M.F. | 6 | 7.5 | 2.3 x 10^-3^ |
| regulation of cytokine production | B.P | 6 | 7.5 | 1.3 x 10^-3^ |
| response to virus | B.P | 6 | 7.5 | 1.2 x 10^-4^ |
| positive regulation of response to stimulus | B.P | 6 | 7.5 | 4.6 x 10^-3^ |
| positive regulation of immune response | B.P | 5 | 6.3 | 4.4 x 10^-3^ |
| regulation of lymphocyte activation | B.P | 5 | 6.3 | 4.9 x 10^-3^ |
| regulation of response to external stimulus | B.P | 5 | 6.3 | 5.8 x 10^-3^ |
| positive regulation of defense response | B.P | 4 | 5.0 | 4.5 x 10^-3^ |
| adaptive immune response | B.P | 4 | 5.0 | 6.9 x 10^-3^ |
| leukocyte mediated immunity | B.P | 4 | 5.0 | 9.1 x 10^-3^ |
| regulation of acute inflammatory response | B.P | 3 | 3.8 | 4.0 x 10^-3^ |
| regulation of B cell mediated immunity | B.P | 3 | 3.8 | 5.6 x 10^-3^ |
| complement activation, classical pathway | B.P | 3 | 3.8 | 8.1 x 10^-3^ |
|  |  |  |  |  |
| ***Cell proliferation/death*** |  |  |  |  |
| regulation of programmed cell death | B.P | 12 | 15.0 | 1.1 x 10^-3^ |
|  |  |  |  |  |
| ***Cell signaling*** |  |  |  |  |
| cell-cell signaling | B.P | 10 | 12.5 | 1.6 x 10^-3^ |
| positive regulation of signal transduction | B.P | 6 | 7.5 | 9.8 x 10^-3^ |
| positive regulation of I-kappaB kinase/NF-kappaB cascade | B.P | 4 | 5.0 | 9.9 x 10^-3^ |
|  |  |  |  |  |
| ***Cell activation*** |  |  |  |  |
| regulation of cell activation | B.P | 5 | 6.3 | 8.8 x 10^-3^ |
|  |  |  |  |  |
| ***Others*** |  |  |  |  |
| extracellular region | C.C. | 23 | 28.7 | 2.6 x 10^-5^ |
| response to wounding | B.P | 16 | 20.0 | 1.1 x 10^-8^ |
| endomembrane system | C.C. | 10 | 12.5 | 7.0 x 10^-3^ |
| positive regulation of multicellular organismal process | B.P. | 7 | 8.8 | 8.8 x 10^-4^ |
| secretion by cell | B.P. | 6 | 7.5 | 2.2 x 10^-3^ |
| protein processing | B.P. | 5 | 6.3 | 1.7 x 10^-3^ |
|  |  |  |  |  |
| ***Pathway*** |  |  |  |  |
| Toll-like receptor signaling pathway | KEGG Pathway | 6 | 7.5 | 4.1 x 10^-4^ |
| systemic lupus erythematosus | KEGG Pathway | 5 | 6.3 | 3.5 x 10^-3^ |
| complement and coagulation cascades | KEGG Pathway | 4 | 5.0 | 9.7 x 10^-3^ |
| # Category: B.P. (biological process); C.C. (cellular component); M.F. (molecular function). | | | | |
