# Supplementary material for: Gene Expression Profiling of Dendritic Cells in Different Physiological Stages under Cordyceps sinensis Treatment
Source: PLoS One. 2012 Jul 19;7(7):e40824. doi: 10.1371/journal.pone.0040824 (PMC3400664; doi:10.1371/journal.pone.0040824)
Supplement: Table S4 — Gene list of A7 group genes analyzed by functional enrichment analysis ( P < 0.05). (DOC) [file pone.0040824.s007.doc]

| **Table S4.** Gene list of A7 group genes analyzed by functional enrichment analysis (*P*< 0.05). | | | | | | |
| --- | --- | --- | --- | --- | --- | --- |
| **Gene description** | **Gene name** | **Entrez ID** | **Loop1 (log2 ratio)** | **Loop2 (log2 ratio)** | **Mean** | **SD** |
| ***enzyme inhibitor activity*** |  |  |  |  |  |  |
| amyloid beta (A4) precursor-like protein 2 | APLP2 | 334 | -0.92 | -0.72 | -0.82 | 0.14 |
| phosphatidylethanolamine binding protein 1 | PEBP1 | 5037 | -0.69 | -0.60 | -0.64 | 0.07 |
| ribonuclease/angiogenin inhibitor 1 | RNH1 | 6050 | -0.64 | -0.84 | -0.74 | 0.14 |
| IQ motif containing GTPase activating protein 1 | IQGAP1 | 8826 | -0.67 | -0.82 | -0.75 | 0.10 |
|  |  |  |  |  |  |  |
| ***small GTPase mediated signal transduction*** |  |  |  |  |  |  |
| dedicator of cytokinesis 1 | DOCK1 | 1793 | -1.30 | -0.79 | -1.05 | 0.36 |
| RAB5C, member RAS oncogene family | RAB5C | 5878 | -0.72 | -0.71 | -0.71 | 0.01 |
| IQ motif containing GTPase activating protein 1 | IQGAP1 | 8826 | -0.67 | -0.82 | -0.75 | 0.10 |
| RAB33A, member RAS oncogene family | RAB33A | 9363 | -0.63 | -0.71 | -0.67 | 0.06 |
|  |  |  |  |  |  |  |
| ***cell surface*** |  |  |  |  |  |  |
| calreticulin | CALR | 811 | -0.66 | -0.63 | -0.64 | 0.02 |
| major histocompatibility complex, class II, DR beta 1 | HLA-DRB1 | 3123 | -1.27 | -1.78 | -1.53 | 0.36 |
| hyaluronan-mediated motility receptor (RHAMM) | HMMR | 3161 | 0.65 | 0.75 | 0.70 | 0.07 |
| phosphatidylethanolamine binding protein 1 | PEBP1 | 5037 | -0.69 | -0.60 | -0.64 | 0.07 |
|  |  |  |  |  |  |  |
| ***oxidative phosphorylation*** |  |  |  |  |  |  |
| ATP synthase, H+ transporting, mitochondrial F0 complex, subunit C3 (subunit 9) | ATP5G3 | 518 | -0.66 | -0.87 | -0.76 | 0.14 |
| ubiquinol-cytochrome c reductase complex (7.2 kD) | UCRC | 29796 | -0.62 | -0.78 | -0.70 | 0.11 |
| ATPase, H+ transporting, lysosomal 34kDa, V1 subunit D | ATP6V1D | 51382 | -0.75 | -0.72 | -0.73 | 0.02 |
|  |  |  |  |  |  |  |
| ***hydrogen ion transmembrane transporter activity*** |  |  |  |  |  |  |
| ATP synthase, H+ transporting, mitochondrial F0 complex, subunit C3 (subunit 9) | ATP5G3 | 518 | -0.66 | -0.87 | -0.76 | 0.14 |
| ubiquinol-cytochrome c reductase complex (7.2 kD) | UCRC | 29796 | -0.62 | -0.78 | -0.70 | 0.11 |
| ATPase, H+ transporting, lysosomal 34kDa, V1 subunit D | ATP6V1D | 51382 | -0.75 | -0.72 | -0.73 | 0.02 |
|  |  |  |  |  |  |  |
| ***SH3 domain binding*** |  |  |  |  |  |  |
| dedicator of cytokinesis 1 | DOCK1 | 1793 | -1.30 | -0.79 | -1.05 | 0.36 |
| glutathione peroxidase 1 | GPX1 | 2876 | -0.73 | -1.22 | -0.98 | 0.34 |
| KH domain containing, RNA binding, signal transduction associated 3 |  | 10656 |  |  |  |  |
|  |  |  |  |  |  |  |
| ***aging*** |  |  |  |  |  |  |
| ATP synthase, H+ transporting, mitochondrial F0 complex, subunit C3 (subunit 9) | ATP5G3 | 518 | -0.66 | -0.87 | -0.76 | 0.14 |
| calreticulin | CALR | 811 | -0.66 | -0.63 | -0.64 | 0.02 |
| phosphatidylethanolamine binding protein 1 | PEBP1 | 5037 | -0.69 | -0.60 | -0.64 | 0.07 |
|  |  |  |  |  |  |  |
| ***antigen processing and presentation of peptide antigen via MHC class I*** |  |  |  |  |  |  |
| calreticulin | CALR | 811 | -0.66 | -0.63 | -0.64 | 0.02 |
| major histocompatibility complex, class II, DR beta 1 | HLA-DRB1 | 3123 | -1.27 | -1.78 | -1.53 | 0.36 |
|  |  |  |  |  |  |  |
| ***oxidative phosphorylation pathway*** |  |  |  |  |  |  |
| ATP synthase, H+ transporting, mitochondrial F0 complex, subunit C3 (subunit 9) | ATP5G3 | 518 | -0.66 | -0.87 | -0.76 | 0.14 |
| ubiquinol-cytochrome c reductase complex (7.2 kD) | UCRC | 29796 | -0.62 | -0.78 | -0.70 | 0.11 |
| ATPase, H+ transporting, lysosomal 34kDa, V1 subunit D | ATP6V1D | 51382 | -0.75 | -0.72 | -0.73 | 0.02 |
