# Supplementary material for: Gene Expression Profiling of Dendritic Cells in Different Physiological Stages under Cordyceps sinensis Treatment
Source: PLoS One. 2012 Jul 19;7(7):e40824. doi: 10.1371/journal.pone.0040824 (PMC3400664; doi:10.1371/journal.pone.0040824)
Supplement: Table S5 — Functional enrichment analysis of A4 group genes by GO-terms and KEGG pathway ( P < 0.05). (DOC) [file pone.0040824.s008.doc]

| **Table S5.** Functional enrichment analysis of A4 group genes by GO-terms and KEGG pathway (*P*< 0.05). | | | | |
| --- | --- | --- | --- | --- |
| **Term** | **Category #** | **Number of genes observed** | **%** | ***P* value** |
| ***Immune response*** |  |  |  |  |
| immune response | B.P. | 13 | 18.5 | 9.3 x 10^-5^ |
| defense response | B.P. | 8 | 11.4 | 2.6 x 10^-2^ |
|  |  |  |  |  |
| ***Cell proliferation/deadth*** |  |  |  |  |
| regulation of cell proliferation | B.P. | 12 | 17.1 | 6.9 x 10^-3^ |
| cell death | B.P. | 9 | 12.8 | 3.6 x 10^-2^ |
| programmed cell death | B.P. | 8 | 11.4 | 4.3 x 10^-2^ |
| negative regulation of programmed cell death | B.P. | 7 | 10.0 | 2.7 x 10^-2^ |
| negative regulation of cell proliferation | B.P. | 7 | 10.0 | 1.8 x 10^-2^ |
| negative regulation of neuron apoptosis | B.P. | 3 | 4.3 | 4.0 x 10^-2^ |
|  |  |  |  |  |
| ***Others*** |  |  |  |  |
| mitochondrion | C.C. | 13 | 18.6 | 9.6 x 10^-3^ |
| response to organic substance | B.P. | 10 | 14.3 | 3.6 x 10^-2^ |
| cellular response to stress | B.P. | 9 | 12.9 | 1.8 x 10^-2^ |
| positive regulation of molecular function | B.P. | 9 | 12.9 | 2.5 x 10^-2^ |
| response to DNA damage stimulus | B.P. | 7 | 10.0 | 2.1 x 10^-2^ |
| DNA repair | B.P. | 6 | 8.6 | 2.3 x 10^-2^ |
| response to nutrient levels | B.P. | 5 | 7.1 | 3.8 x 10^-2^ |
| response to extracellular stimulus | B.P. | 5 | 7.1 | 4.7 x 10^-2^ |
| double-strand break repair | B.P. | 4 | 5.7 | 5.9 x 10^-3^ |
| DNA recombination | B.P. | 4 | 5.7 | 2.1 x 10^-2^ |
| regulation of membrane potential | B.P. | 4 | 5.7 | 2.1 x 10^-2^ |
| JAK-STAT cascade | B.P. | 3 | 4.3 | 2.1 x 10^-2^ |
| cellular response to oxidative stress | B.P. | 3 | 4.3 | 2.3 x 10^-2^ |
| neutral lipid metabolic process | B.P. | 3 | 4.3 | 3.7 x 10^-2^ |
| neuromuscular process | B.P. | 3 | 4.3 | 4.0 x 10^-2^ |
| positive regulation of transcription factor activity | B.P. | 3 | 4.3 | 4.3 x 10^-2^ |
| organic ether metabolic process | B.P. | 3 | 4.3 | 4.6 x 10^-2^ |
| protein heterodimerization activity | M.F. | 5 | 7.1 | 2.4 x 10^-2^ |
| transcription coactivator activity | M.F. | 5 | 7.1 | 3.1 x 10^-2^ |
|  |  |  |  |  |
| ***Pathway*** |  |  |  |  |
| Tryptophan metabolism | KEGG | 4 | 5.7 | 3.1 x 10^-3^ |
| # Category: B.P. (biological process); C.C. (cellular componet); M.F. (molecular function). | | | |  |
