# Supplementary material for: Gene Expression Profiling of Dendritic Cells in Different Physiological Stages under Cordyceps sinensis Treatment
Source: PLoS One. 2012 Jul 19;7(7):e40824. doi: 10.1371/journal.pone.0040824 (PMC3400664; doi:10.1371/journal.pone.0040824)
Supplement: Table S6 — Primers for real-time PCR. (DOC) [file pone.0040824.s009.doc]

| **Table S6.** Primers for real-time PCR. | | | |
| --- | --- | --- | --- |
| **Gene symbol** | **Entrez ID** | **Forward primer** | **Reverse primer** |
| ATP5G3 | 518 | TTGGCAGCCTTATCATTGGTT | TGAGAACAGCTGCTGCTTCAG |
| ATP6V1D | 51382 | CGGGCAAAGACCGAATTG | TTTAAACGAGCCTTCATGATGGT |
| CAT | 847 | CTGGAGAAGTGCGGAGATTCA | AATGCCCGCACCTGAGTAAC |
| CCL18 | 6362 | CCAATAAGAAGTGGGTCCAGAAA | GCCCTCGCAGCTTCCA |
| CCL22 | 6367 | CCAGAAGCCTGTGCCAACTC | ACAGCACGGAGGTGACCAA |
| CCL7 | 6354 | AGAAAACCCAAACTCCAAAGCTT | TTGTTTCTCAAGTCATGGCTTGTT |
| CD83 | 9308 | AGGCCTCGAAAACCATCACA | GGTGGCCATGGAGAAGCA |
| CYP1B1 | 1545 | TGTGCCTGTCACTATTCCTCATG | GGGAATGTGGTAGCCCAAGA |
| HLA-DRB1 | 3123 | TGGCAGCGCCTCATCTTC | CGGGAGGCCATACGGTTT |
| INDO | 3620 | CAGCGCCTTGCACGTCTAG | CCTTTGCCCCACACATATGC |
| KYNU | 8942 | TGATCTAGCACATGCAGTTGGAA | TAGGAACACCAGCAGGCAAAA |
| UCRC | 29796 | TGGGCGTCATGTTCTTCGA | CTCGTTGATGTGGTCGTAGATAGC |
| WARS | 7453 | TCCAGTGCCTTATCCCATGTG | GCGACGTCCCTTGTCATTCT |
